# Introduction of a New Mandibular Reconstruction Procedure Using a Sagittal Split: A Case Report

**DOI:** 10.7759/cureus.70271

**Published:** 2024-09-26

**Authors:** Han-Pang Liu, Chi-Sheng Cheng, Chien-Ming Chang

**Affiliations:** 1 Department of Stomatology, Oral and Maxillofacial Surgery, Taichung Veterans General Hospital, Taichung, TWN; 2 Department of Oral and Maxillofacial Surgery, Chang Bing Show Chwan Memorial Hospital, Changhua, TWN

**Keywords:** oral malignant tumor, sagittal mandibular split, segmental mandibular resection, soft tissue excision, vascularized forearm flap

## Abstract

Soft tissue excision and segmental mandibular resection for the treatment of benign or malignant oral tumors result in surgical defects of varying extents. These procedures are often followed by adjuvant chemotherapy and radiotherapy, which induce further adverse events with limited available treatment options. To reduce the morbidity and enhance the success rate of mandibular reconstruction, we developed a novel technique that combines a sagittal mandibular split and the use of a vascularized forearm flap graft. Here, we describe our experience with this procedure in an older male patient. The bridging bone segment was pedicled using the mylohyoid muscle and periosteum, and a reliable vascularized forearm graft was used to repair the soft tissue defect. The patient experienced a rapid recovery, and a two-year follow-up revealed that the bone and skin grafts tolerated radiotherapy well. We conclude that this technique is a viable alternative for patients with a bony gap around 4 cm or in whom a vascularized fibular flap graft is contraindicated.

## Introduction

Oral and mandibular defects resulting from the resection of benign or malignant oral tumors, trauma, osteonecrosis of the jaw, and osteomyelitis have long presented a challenge to oral surgeons. Currently, mandibular reconstruction strategies are often combined with procedures intended to restore the oral mucosa, facial skin, mandibular bone, and dentition, with the ultimate goals of maintaining the facial profile, continuity of the oral commissure, and mastication ability. Particularly, mandibular reconstruction after the resection of a malignant oral tumor is frequently associated with some losses of mucosal and soft tissues. Moreover, radiotherapy is often initiated within six weeks after surgery and must therefore be considered during the reconstruction process.

Currently, reconstruction plates, non-vascularized bone grafting, vascularized flaps, and bone graft substitutes are used for mandibular reconstruction. Of these, non-vascularized bone grafts and particulate marrow and cancellous bone (PMCB) grafts yield higher delayed reconstruction success rates, as failures are often attributed to the inability of a graft to withstand intraoral contamination [1].

Vascularized bone grafts are considered advantageous because they enable the immediate and simultaneous reconstruction of soft and bony tissues. These grafts can also be placed in irradiated tissues, and implants can be placed during the same procedure. However, the donor site morbidity associated with a vascularized bone flap is considerably higher. Moreover, although a reconstruction plate and vascularized flap can tolerate the irradiation associated with the treatment of a malignant oral tumor, the compromised soft tissues and scar contracture often lead to plate exposure [2].

In summary, no single method of reconstruction can address all the potential variables affecting a patient with a mandibular defect, particularly as reconstruction after treatment for oral cancer (e.g., oral squamous cell carcinoma (OSCC)) is challenged by factors such as previous irradiation of the surgical site or the high risk of postoperative adjuvant radiotherapy. In this report, we describe our experience with a novel technique that combines a sagittal mandibular split and the use of a vascularized forearm flap graft in an older patient undergoing treatment for OSCC.

## Case presentation

A 63-year-old man, with a medical history of diabetes mellitus, presented with an OSCC of the lower left posterior gingiva with invasion to the mandibular bone, stage cT4aN0M0. The patient was completely edentulous and had been fitted with a complete denture that had not been worn for a long time. The patient’s treatment plan included wide excision, segmental mandibulectomy, ipsilateral supraomohyoid neck dissection, and immediate mandibular reconstruction. Following discussion, the patient agreed and signed the informed consent to undergo a procedure involving sagittal splitting of the mandible and a free radial forearm flap for soft and hard tissue reconstruction, respectively.

Surgical mandibular reconstruction via sagittal split osteotomy (SSO)

**Figure 1:**
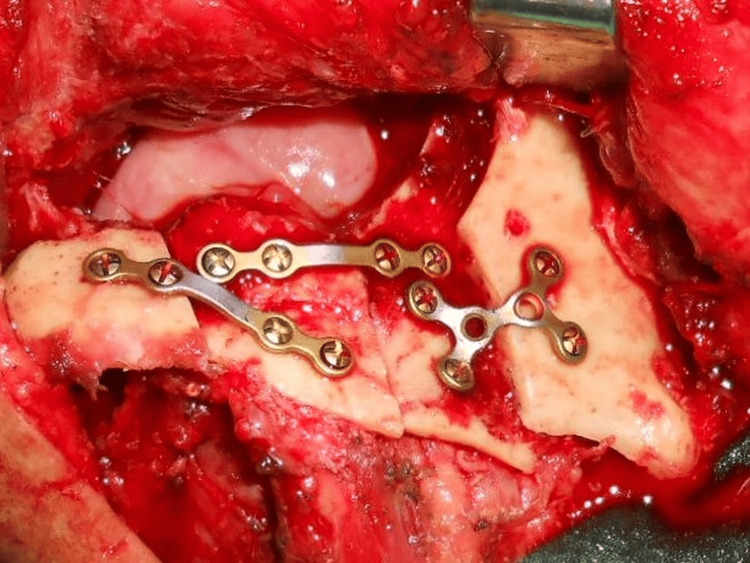
Intraoperative photo Using sagittal split osteotomy to reconstruct the segmental defect of the mandible. Muscle attachments of the bone segments remained intact.

Intraoperatively, after tumor ablation, the surgical defect included the buccal mucosa and part of the floor of the mouth, as well as a 4-cm bone defect from the parasymphysis to the ascending ramus. Sagittal split osteotomies by using osteotomes and a piezoelectric knife of the symphysis remnant and left ramus were performed. Both inner bony segments were advanced to achieve mandibular continuity. The lingual soft tissue and periosteum were not released to avoid reducing the blood supply from the myohyoid muscle and periosteum. The bony segments were fixed with 2.0-mm titanium miniplates (Figure 1). Next, a 6 cm × 8 cm radial forearm free flap was harvested and used to cover the surgical defect, as sufficient surrounding soft tissue for approximation was not available. No flap necrosis, soft tissue dehiscence, or fistula was observed at the surgical site perioperatively. Oral intake was reintroduced on the 10th day after surgery, following the removal of the nasogastric tube. The patient was then discharged without any complications at the surgical sites.

Follow-up

**Figure 2:**
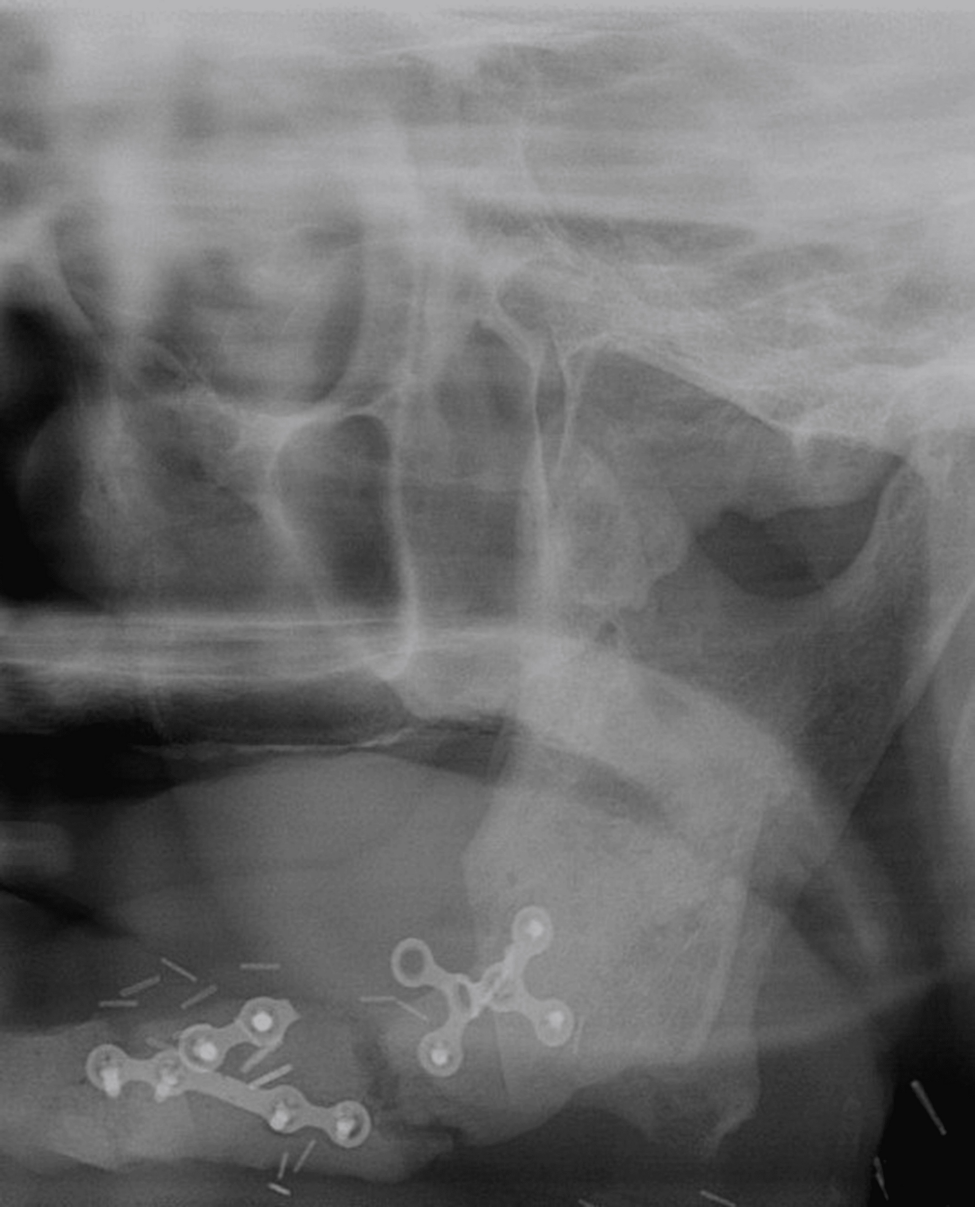
Panoramic radiography of a two-year postoperative follow-up Mandibular continuity was maintained for two years postoperatively. However, three screws and half of a plate were removed during the follow-up period due to loosening. New bone formation was observed, successfully connecting the bone segments.

**Figure 3:**
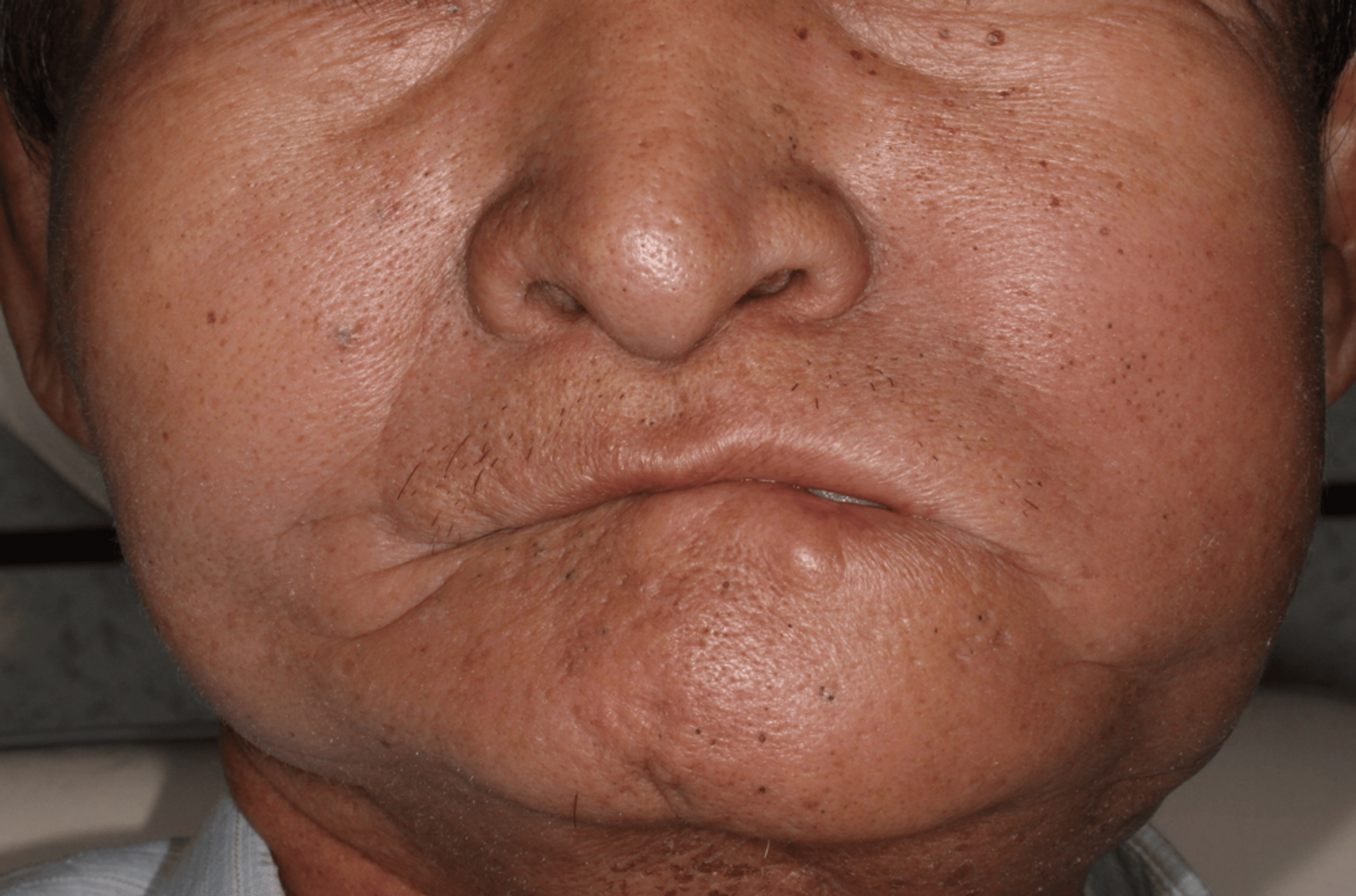
Extraoral photo of a two-year postoperative follow-up No facial asymmetry or deviation of the chin was noted after radiotherapy.

**Figure 4:**
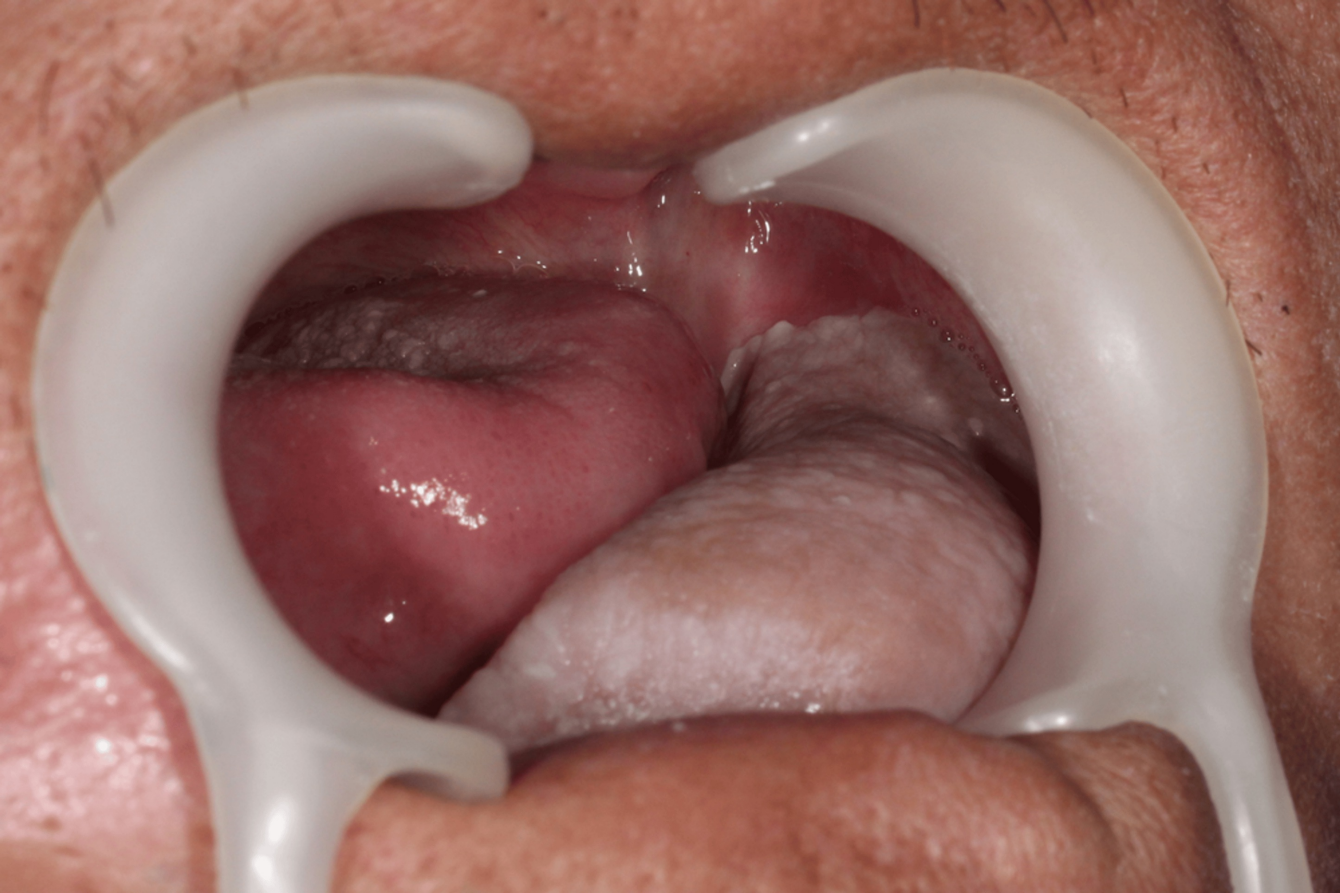
Intraoral photo of a two-year postoperative follow-up No bony exposure was noted, and the tissue volume remained sufficient after radiotherapy.

Postoperatively, the patient received adjuvant radiotherapy at a total dose of 6,600 cGy in 33 fractions. The adverse events observed during a two-year follow-up were Grade I mucositis and loosening of three screws. No bone exposure or fistula was noted, and the soft tissue volume remained adequate after scar contracture (Figures 2-4).

## Discussion

According to previous studies, immediate mandibular reconstruction provides a better quality of life than delayed reconstruction [3-6]. In our case, the patient exhibited a moderately sized soft and hard tissue defect, and therefore primary reconstruction was considered. However, this case involved clinical stage IVa disease with a high likelihood of postoperative adjuvant radiotherapy, and a non-vascularized bone block graft and PMCB graft were contraindicated. Therefore, immediate mandibular reconstruction with a titanium plate or vascularized free bone flap appeared to be a reasonable solution for our patient.

Titanium plates provide a safe and rapid option for mandibular reconstruction after resection surgery for oral cancer [6]. In 1991, Gullane noted that, in such cases, the radiation dose at the bone-plate interface increased by only 15% within a 1.1-mm margin of the surrounding tissue [7]. However, this reconstruction procedure is associated with a high risk of complications, and some studies even observed that the inclusion of adjuvant radiotherapy increases the probability of complications such as infection, plate exposure, and plate failure [8].

According to the algorithm developed by Takushima et al. [9], our case was classified as an “L” defect with a mucosal defect. Therefore, a vascularized flap seemed to be a reasonable solution for reconstruction. Although a fibula flap is the best option for a vascularized graft, the length of the bone defect in this patient was only 4 cm, and therefore it was not necessary to sacrifice a fibula (which can provide up to 26 cm of bone), particularly given the risk of postoperative ankle stiffness or leg numbness. Alternatively, a vascularized radial forearm flap with radial tissue could be used, although this option is complicated by a postharvest radial fracture rate of 23-42% [10]. Our novel technique, which combines SSO with a vascularized radial forearm flap graft, can be used to repair the mandibular continuity and the mucosal defect simultaneously and is associated with a relatively lower level of morbidity.

Previously, Hell [11] applied SSO with primary wound closure for the treatment of an OSCC of the anterior floor of the mouth with deep invasion to the bony chin. In that case, soft tissue dehiscence was observed a few days after surgery and was repaired using a pedicled nasolabial flap. That patient did not undergo postoperative radiotherapy, and a re-exposure operation performed 16 months later revealed complete bone healing. Both our case and the case reported by Hell indicate that the SSO technique can be applied appropriately to an edentulous patient with a defect length <4 cm. Moreover, our observations over a two-year follow-up period demonstrate the subsequent tolerability of the surgical site to postoperative radiotherapy.

The primary limitation of this technique is the size of the bony defect. According to our measurements, if the contralateral SSO can be used, bone advancement can reach up to 6 cm. A midline defect of less than 4 cm can be repaired using bilateral SSO. However, for defects larger than 6 cm, a vascularized fibular flap is the most suitable option for reconstructing the bone gap. While dental implant placement may not initially be feasible, if postoperative radiotherapy is not required, a staged approach guided bone regeneration with a particulate bone graft can be considered to build up the ridge for future dental implants

## Conclusions

In conclusion, our newly developed technique, combining SSO with a vascularized radial forearm flap graft, offers a promising alternative for patients with bone defects around 4 cm, particularly when a vascularized fibular flap is contraindicated (e.g., due to a history of peripheral artery disease or leg trauma). A two-year follow-up demonstrated that both the bone and skin remained healthy, making this method a suitable option for patients who are likely to receive post-operative radiotherapy.
